# Complications of intubation in an academic medical-surgical ICU

**DOI:** 10.1186/2197-425X-3-S1-A431

**Published:** 2015-10-01

**Authors:** K Shore, S Walker, L Palozzi, SE Lapinsky

**Affiliations:** University of Toronto, Critical Care, Toronto, Canada; Mount Sinai Hospital, Critical Care, Toronto, Canada

## Introduction

Endotracheal intubation is one of the highest risk interventions taking place in the intensive care unit (ICU) and can result in significant complications. In many academic centres, endotracheal intubation is frequently performed by junior trainees under supervision. There is a lack of data to support the safety of this approach and whether there is an association between complications and provider experience in the ICU setting.

## Objectives

1) To identify factors associated with complications from endotracheal intubation in the ICU.

2) To evaluate the impact and risk of endotracheal intubations performed by junior trainees in the ICU.

## Methods

We conducted a retrospective review of intubation practices in the 16-bed ICU at Mount Sinai Hospital in Toronto, Canada, from 2011 to 2013 using a quality improvement database. Complications included hypoxemia, hypotension, bradycardia, aspiration and cardiac arrest. Severe complications were defined as new SpO2 < 80%, SBP < 70 or MAP < 50 mmHg, or cardiac arrest. Categorical data was compared using either the *X*^2^ or Fisher's exact test. All reported P values are two-sided and considered significant when P < 0.05. Analyses were performed using STATA statistical software.

## Results

239 intubations were analyzed. Forty percent of intubations were performed by residents in post-graduate year 1 to 3, the majority being Internal Medicine trainees. The success rate by this group was 60% compared with 93% for fellows and attending physicians (p < 0.001). The number of intubation attempts was higher for residents (median of 2 [IQR 1-3]) compared with all other operators (median of 1 [IQR 1-2]). The overall complication rate was 58.2% with severe complications in 16.7%. Residents and fellows had similar complication rates (63.5% and 54.2%, p = 0.43). The complication rate correlated with increasing number of attempts (p = 0.01), but not with Cormack and Lehane airway grade (p = 0.56), timing of intubation (weekday vs. on call, p = 0.78), or use of sedation (p = 0.64). Only 9% of patients in the cohort received neuromuscular blocking agents for intubation. The use of video laryngoscopy was associated with a non-significant trend towards improved success (77 vs 93%, p= 0.19) and complication rate (59 vs 50%, p = 0.23).

## Conclusions

Endotracheal intubation by junior trainees in an academic ICU is safe, but the low success rate by junior trainees suggests that timely access to backup support is necessary. Efforts should be made to minimize attempts, which may include restricting attempts taken by junior trainees and increasing training and utilization of video laryngoscopy. A quality improvement database is of value in monitoring this high-risk procedure in the ICU.

